# Gender stereotype endorsement differentially predicts girls' and boys' trait-state discrepancy in math anxiety

**DOI:** 10.3389/fpsyg.2015.01404

**Published:** 2015-09-17

**Authors:** Madeleine Bieg, Thomas Goetz, Ilka Wolter, Nathan C. Hall

**Affiliations:** ^1^Department of Empirical Educational Research, University of KonstanzKonstanz, Germany; ^2^Department of Empirical Educational Research, Thurgau University of Teacher EducationKreuzlingen, Switzerland; ^3^Instrument Development and Research, Leibniz Institute for Educational TrajectoriesBamberg, Germany; ^4^Department of Educational and Counselling Psychology, McGill UniversityMontreal, QC, Canada

**Keywords:** anxiety, mathematics, self-concept, gender stereotype, trait anxiety, state anxiety, ecological momentary assessment, diary study

## Abstract

Mathematics is associated with anxiety for many students; an emotion linked to lower well-being and poorer learning outcomes. While findings typically show females to report higher trait math anxiety than males, no gender differences have to date been found in state (i.e., momentary) math anxiety. The present diary study aimed to replicate previous findings in investigating whether levels of academic self-concept was related to this discrepancy in trait vs. state anxiety measures. Additionally, mathematics-related gender stereotype endorsement (mathematics is a male domain) was investigated as an additional predictor of the trait-state discrepancy. The sample included 755 German 9th and 10th graders who completed self-report measures of trait math anxiety, math self-concept, and gender stereotype endorsement, in addition to state measures of anxiety after math classes by use of a standardized diary for 2–3 weeks (*N*_within_ = 6207). As expected, females reported higher trait math anxiety but no gender differences were found for state math anxiety. Also in line with our assumptions, multilevel analyses showed the discrepancy between trait and state anxiety to be negatively related to students' self-concept (i.e., a lower discrepancy for students with higher self-concepts). Furthermore, gender stereotype endorsement differentially predicted the trait-state discrepancy: When controlling for self-concept in mathematics, females who endorsed the gender stereotype of math being a male domain more strongly overestimated their trait math anxiety as compared to their state anxiety whereas this effect was not significant for males. The present findings suggest that gender stereotype endorsement plays an important role in explaining gender differences in math anxiety above and beyond academic self-concept. Implications for future research and educational practice are discussed.

## Introduction

Mathematics is a domain of high importance, given the need for basic mathematical competencies in many degree programs and professional careers, making the frequent reports of math anxiety by students a cause for concern (Goetz et al., 2004, 2014). In general, greater anxiety has been found to contribute to lower well-being (Diener, 2000) and poorer achievement outcomes, as well as lower long-term academic motivation and use of more superficial learning strategies (Pekrun et al., 2002). Research further shows higher levels of math anxiety to negatively predict decisions in favor of course enrollment and career choices in math-intensive fields (Eccles, 1985; Wigfield et al., 2002; Perez et al., 2014).

Meta-analyses consistently show female students to report higher levels of mathematics anxiety (e.g., Else-Quest et al., 2010), with studies additionally showing female students typically report lower self-concepts in mathematics relative to male students (Hyde et al., 1990; Goetz et al., 2008, 2013). These gender differences are, however, in direct contrast to studies showing gender differences in mathematics performance to be notably small or non-existent (Hyde et al., 2008; Else-Quest et al., 2010), and instead appear to reflect long-held stereotypes that female students are less capable in this domain relative to their male counterparts (Steffens et al., 2010; Steffens and Jelenec, 2011). This troubling persistence of gender stereotypes and females' negative attitudes toward mathematics is further assumed to contribute to the underrepresentation of females in many STEM domains in which mathematical competencies are a prerequisite (Watt, 2006; Halpern et al., 2007).

With respect specifically to math anxiety, previous research has been almost exclusively based on self-reports of trait-like anxiety as opposed to state assessments utilizing real-time measures. We define trait and state math anxiety from a methodological perspective (see Bieg et al., 2013). Reports of trait math anxiety reflect (mentally) generalized levels of anxiety across different time points in math-related situations. In contrast, reports of state math anxiety reflect levels of momentary anxiety in real-life math-related situations. Thus, the primary difference between trait and state math anxiety, as we define it, is the different level of generalization across time (see Pekrun, 2006). The ecological validity of such trait-based measures regarding their potential to capture emotions as they are experienced has been questioned due to their reliance on retrospective or global reports and resulting susceptibility to recall bias (Scollon et al., 2003). Although both trait and state measures are assumed to assess anxiety, these assessment methods can lead to very different results with respect to the degree to which specific emotions are reported (Bieg et al., 2014). Thus, an intriguing question remains as to the extent to which the gender differences observed on self-report trait measures of habitual math anxiety reflect actual gender differences in the lived experience of anxiety in the mathematics domain.

One recent study found gender differences in students' trait but not state mathematics anxiety, with girls reporting higher levels than boys on trait measures, but not on the state measures (Goetz et al., 2013). Emotion levels are also typically found to be higher on trait assessments as compared to state measures (intensity bias; Buehler and McFarland, 2001; Levine et al., 2006). Further, such discrepancies between trait self-reports and state anxiety measures—differences that could be interpreted as a measure for the discrepancy between perceived as compared to actual anxiety—has been found to be largely explained by students' perceptions of competence (e.g., academic self-concept; Goetz et al., 2013; Bieg et al., 2014) underscoring the potential for trait assessments to be more strongly biased by subjective beliefs (Robinson and Clore, 2002). Accordingly, individuals' beliefs regarding gender stereotypes can also be assumed to influence trait reports more than state reports of emotions, with the endorsement of math-related gender stereotypes likely playing a role, particularly for females, in the trait-state discrepancy. The role of math-related gender stereotypes as moderators of the trait-state discrepancy in students' math anxiety, however, to date remains unexplored.

## Theoretical background

### Math as gender-stereotyped domain

Mathematics has long been viewed as a typically male domain; an assumption that continues to be observed in research on gender stereotypes in educational settings (Plante et al., 2009; Steffens et al., 2010; Cvencek et al., 2011; Steffens and Jelenec, 2011; Passolunghi et al., 2014). Stereotypes are defined as the attributions people make regarding the abilities and characteristics of members of a certain group and assumptions about how members of the stereotyped group typically behave (Ruble et al., 1984; Eagly et al., 2000). Although stereotypes are assumed to facilitate human behavior and decision-making in complex environments, they nonetheless are consistently found to have negative effects for the stereotyped group (e.g., stereotype threat effect; Steele and Aronson, 1995; Schmader et al., 2004; Maloney et al., 2013). With respect to the domain of mathematics, research shows males to hold stronger gender stereotypes than females (e.g., Hyde et al., 1990; Rowley et al., 2007) with the endorsement of math-related gender stereotypes found to predict more negative attitudes regarding math ability, and possibly lower involvement in mathematics-related professions, for female students (Eccles, 1994; Schmader et al., 2004; Kurtz-Costes et al., 2008). Research shows multiple sources can contribute to the formation of gender stereotypes in mathematics such as the math anxiety and beliefs of female teachers and parents (Beilock et al., 2010; Gunderson et al., 2012).

### Gender differences in math-related attitudes and anxiety

Meta-analyses of international research typically show small or no gender differences in math performance (Else-Quest et al., 2010; Lindberg et al., 2010; Hyde, 2014; Voyer and Voyer, 2014). Research on math-related attitudes, however, has found individuals' attitudes regarding mathematics to vary to a much higher degree, with girls tending to report less positive attitudes (Watt, 2004; Nagy et al., 2008) and higher levels of anxiety regarding mathematics than boys (Hyde et al., 1990; Else-Quest et al., 2010). It is important to note that most of this research has investigated mathematics anxiety using trait assessments that, as noted above, may not as accurately reflect *in-situ* emotional experiences as would state assessments.

### Assessment of students' math anxiety and the accessibility model of emotional self-report

There are various possible methods for assessing students' math anxiety. Most typically, students are asked about their math anxiety “in general” as a measure of habitual or trait anxiety, with more ecologically valid momentary or state assessments tending to be underutilized (Scollon et al., 2009; Schwarz, 2012). In their accessibility model of emotional self-report, Robinson and Clore (2002) attempt to account for this discrepancy in ecological validity in suggesting that trait assessments of emotional self-reports are more strongly influenced by semantic memory (subjective beliefs, stereotypic beliefs). According to the theory, it can be assumed that because of the transient nature of emotions, it is not possible to directly retrieve emotions from memory. However, when one is asked about emotions “in general,” there is a process of evaluation and aggregation that occurs. It is at this point where recall biases and subjective beliefs may come into play. In contrast, state assessments are more direct in nature and therefore assumed to be less prone to influences by personal beliefs and recall biases, with several studies having provided empirical evidence in support of this assumption (Barrett, 1997; Robinson and Clore, 2002).

### Moderators of the trait-state discrepancy

Recent research shows mean levels of emotion reports to differ substantially between trait and state measures, with trait emotion levels in mathematics (including anxiety) being usually higher than those for state emotions (Bieg et al., 2014). A recent study by Goetz et al. (2013) with 5th to 10th graders found gender differences in trait math anxiety (lower levels for boys) but not in state math anxiety. Furthermore, the discrepancy between trait and state assessments, which could be interpreted as the comparison of retrospective perceptions to actual experiences of anxiety, was largely accounted for by students' self-concept in mathematics such that students' with higher self-concepts were found to have a lower trait-state discrepancy in math anxiety.

In addition to identity-related beliefs such as self-concept, gender stereotypes are explicitly addressed in Robinson and Clore's (2002) accessibility model as an element of semantic memory that can bias trait self-report measures. Accordingly, the gender stereotype in mathematics can be assumed to play a role in the trait-state discrepancy in math anxiety in influencing students' reports on trait anxiety measures more so than on state anxiety measures. For this reason, students' endorsement of a math-related gender stereotype is assumed to be an additional significant predictor of the trait-state discrepancy, with the effects of this stereotype expected to differ for males as compared to females. As the stereotype favors boys, girls who endorse the stereotype should show a higher trait-state discrepancy in math anxiety. For boys, however, this relation should be the inverse: Boys who more strongly endorse this stereotype should report lower trait anxiety resulting in a smaller trait-state math anxiety discrepancy.

### The present research

Following from recent findings on potential moderators of the discrepancy between trait and state emotion measures, our study aimed to examine an additional predictor of this trait-state discrepancy in the context of gender differences in math anxiety. Assuming that trait measures reflect individuals' *beliefs* about emotions, whereas state measures are assumed to better reflect individuals' *actual* emotions, the trait-state discrepancy can be understood to indicate the extent to which generalized perceptions of one's emotions differ from one's real-life and *in-situ* emotional experiences. It was expected that study findings would replicate previous study (Goetz et al., 2013) with gender differences in trait math anxiety as compared to state math anxiety being moderated by self-concept levels. As higher levels of perceived control have consistently been found to predict lower anxiety (Pekrun, 2006), students' with higher self-concepts were similarly expected to report lower trait anxiety levels, resulting in a lower trait-state discrepancy.

Additionally, the present study examined students' endorsement of a math-related gender stereotype as a predictor of this trait-state discrepancy. It was assumed that higher levels of stereotype endorsement would bias girls' trait self-reports differently than those for boys, given the negative performance implications of this stereotype for girls as opposed to the positive implications for boys. It was therefore anticipated that girls' trait-state discrepancy in math anxiety would be significantly higher than the trait-state discrepancy observed for boys.

To summarize, based on the results of Goetz et al. (2013), gender differences were expected in mathematics anxiety on trait-oriented self-report measures but not on state assessments during mathematics instruction (replication; Hypothesis 1). We further expected to find a previously observed discrepancy in trait vs. state assessments, with this discrepancy explained to a significant extent by students' mathematics self-concept (replication; Hypothesis 2). Additionally, students' endorsement of a mathematics-related gender stereotype was expected to correspond with a stronger trait-state discrepancy for females, with males who endorsed the stereotype showing lower discrepancy levels (extension; Hypothesis 3).

## Material and methods

### Ethical statement

Prior to participating in the study, the teachers, students, and parents (depending on school regulations) were informed about study contents and procedure. Confidentiality of data was guaranteed and participation was voluntarily such that withdrawal from the study was possible at any time. Data were collected confidentially and all information that could link individual participants to their results was destroyed before analyzing the data.

### Participants and procedure

German students (*N* = 755; 55.1 % female) from 42 classes of grades 9 and 10 (*M*_age_ = 15.7, *SD* = 0.72) of the highest academic track (Gymnasium, about one third of the total student population; Federal Statistical Office, 2015) participated in a diary study in the domain of mathematics. Students completed an initial paper-and-pencil questionnaire including trait measures of anxiety and related constructs (e.g., mathematics self-concept), as well as demographic items, administered by trained experimenters. Students subsequently participated in a 2–3-week study period during which they completed self-report measures addressing the study variables after each mathematics lesson as part of a short questionnaire. This protocol resulted in *N* = 6207 entries in the standardized diary with a mean of *M* = 8.22 entries per student. Students who rated only one lesson, or completed more than one standardized diary questionnaire per lesson, were excluded from the analyses (resulting in an exclusion of 5 students).

### Trait variables

To allow for trait and state anxiety ratings to be comparable, *trait mathematics anxiety* was assessed using two items obtained from a larger five-item scale (based on PALMA; Pekrun et al., 2007) that were formulated in parallel to the state assessment (e.g., “During mathematics instruction I often feel anxious”). The two items had satisfactory reliability (trait questionnaire: Spearman-Brown ρ = 0.73). The anxiety score summing across the two items was highly correlated with the original five-item scale (*r* = 0.91). Although assessing constructs with more items may be preferable with respect to reliability and validity, empirical evidence nonetheless suggests that measures having fewer items can be sufficiently valid (Gogol et al., 2014). *Self-concept in mathematics* was assessed using three items from the Self Description Questionnaire II (SDQ; Marsh, 1990; α = 0.88; e.g., Mathematics is one of my best subjects), and students' endorsement of the *gender stereotype* (“Math is a male domain”) was assessed using a single-item measure (adapted from Fennema and Sherman, 1977; Hyde et al., 1990). Trait mathematics anxiety items, self-concept items, and stereotype endorsement were rated on a five-point Likert scale ranging from 1 = *strongly disagree* to 5 = *strongly agree*.

### State anxiety

State mathematics anxiety (Spearman-Brown ρ = 0.71) was assessed using two items formulated in parallel to the trait mathematics anxiety items with respect to both phrasing and response format (e.g., “In this lesson I felt anxious”; 1 = *strongly disagree* to 5 = *strongly agree*). The parallel formulation of the trait and state measures ensured the comparability of anxiety levels required to examine our research question regarding potential trait-state discrepancies.

### Statistical analyses

Study data were analyzed with hierarchical linear modeling to account for multiple measurement points per student and students nested in classes (three levels). First, we combined the anxiety measures (trait and state) to assess them as a single dependent variable in the hierarchical linear regression models. To separate the trait from the state anxiety measures, we created a dichotomous dummy variable representing the method used to assess students' anxiety (“*Trait*”: 0 = state, 1 = trait). In the multilevel regression models, the effect of this dummy variable can be interpreted as the magnitude of the difference between trait self-reports vs. state self-reports of anxiety: the trait-state discrepancy. To test the predictive validity of self-concept (Model 1) and gender stereotype endorsement (Model 2) of this discrepancy in girls and boys, we sequentially introduced the two variables (*z*-standardized) in the hierarchical linear models resulting in the following model equations.

Model 1 multilevel equation:

*Y*_ijk_[Emotion value *i* of student *j in class k*] = γ_000_ + γ_100_(Trait) + γ_010_(*z*Self-concept) + γ_110_(*z*Self-concept ∗ Trait) + r_0_ + r_1_(Trait) + u_00_ + e

Model 2 multilevel equation (notation as in **Table 2**):

*Y*_ijk_[Emotion value *i* of student *j in class k*] = γ_000_ + γ_100_(Trait) + γ_020_(*z*Stereotype endorsement) + γ_120_(*z*Stereotype endorsement ∗ Trait) + r_0_ + *r*_1_(Trait) + u_00_ + e

In the multilevel models, self-concept and stereotype endorsement each function as a predictor of the slope of the Trait dummy variable (slope-as-outcome model) resulting in a cross-level interaction between Level 1 and Level 2 (Self-concept × Trait interaction; γ_110_and Stereotype endorsement × Trait interaction; γ_120_). The magnitude of these interaction terms therefore indicates the effect of self-concept or stereotype endorsement on the trait-state discrepancy, respectively. Positive effects indicate that higher self-concept/stereotype values are associated with greater discrepancies between trait and state assessments, whereas negative effects indicate smaller discrepancies. For the sake of completeness, self-concept and stereotype endorsement were both included in each model as a predictor of the intercept (γ_010_). These effects however, were not a primary concern with respect to our study hypotheses.

Additionally, our analyses included both self-concept and gender stereotype endorsement as predictors in a single model (Model 3):

*Y*_*ijk*_[Emotion value *i* of student *j in class k*] = γ_000_ + γ_100_(Trait) + γ_010_(*z*Self-concept) + γ_020_(*z*Stereotype endorsement) + γ_110_(*z*Self-concept ∗ Trait) + γ_120_(*z*Stereotype endorsement ∗ Trait) + γ_130_(*z*Self-concept ∗ *z*Stereotype endorsement ∗ Trait) + r_0_ + r_1_(Trait) + u_00_ + e

As we were primarily interested in the strength of the effects of the predictors on the trait-state discrepancy in math anxiety, that for stereotype endorsement were assumed to be reversed depending on a student's gender, Models 1–3 were analyzed separately for boys and girls (see Supplementary Material for hierarchical linear regression models for complete sample).

## Results

### Descriptives

The intraclass correlation coefficient (with respect to Levels 1 and 2) for the state anxiety measures was ICC(1) = 0.32 for female students and ICC(1) = 0.34 for male students. In addition, we calculated the ICC(2) that can be interpreted as a reliability measure of the aggregated state value (Lüdtke et al., 2006). The ICC(2) was 0.79 for girls and 0.81 for boys, indicating sufficient reliability for the aggregated state values. To examine our research questions (Hypothesis 1), mean-level differences were evaluated in the first step, showing gender differences in mathematics anxiety for trait anxiety (Cohen's *d* = −0.12) but not for state anxiety measures (see Table 1). Furthermore, male and female students were found to differ significantly in their math-related self-concepts, with boys reporting higher self-concept levels. Males were also found to more strongly endorse the math gender stereotype. The correlation between math self-concept and gender stereotype endorsement was *r* = −0.36 for girls and *r* = 0.18 for boys.

**Table 1:** **Descriptive statistics and mean level differences**.

**Scales**	**Girls**		**Boys**		***t*-value**	**Effect size *d***
	***M***	***SD***	***M***	***SD***		
Anxiety-Trait	1.87	0.90	1.72	0.84	−2.32^*^	−0.12
Anxiety-State	1.65	0.84	1.64	0.81	−0.32	−0.01
Self-concept	2.86	1.18	3.26	1.15	5.19^***^	0.24
Stereotype Endorsement	1.76	1.14	2.12	1.24	4.39^***^	0.21

*Positive t-values reflect higher scores for boys. For multi-item measures, scale values were divided by the number of items. n = 416 girls, n = 339 boys*.

* *p < 0.05*,

*** *p < 0.001*.

### Main analyses

For the main analyses, hierarchical linear models comprising 3 levels (measurement points at Level 1, students at Level 2, and classes at Level 3) were calculated to evaluate Hypotheses 2 and 3 (see Table 2). Analyses were conducted separately for each gender to more explicitly evaluate the anticipated differential effects for girls vs. boys.

**Table 2:** **Hierarchical linear regression models with anxiety as dependent variable**.

	**Girls**			**Boys**		
	**Model 1**	**Model 2**	**Model 3**	**Model 1**	**Model 2**	**Model 3**
**LEVEL 1**						
Intercept (γ_000_)	1.68^***^ (0.04)	1.68^***^ (0.04)	1.68^***^ (0.04)	1.67^***^ (0.04)	1.66^***^ (0.04)	1.67^***^ (0.04)
Trait (γ_100_)	0.22^***^ (0.04)	0.22^***^ (0.05)	0.22^***^ (0.04)	0.07 (0.05)	0.06 (0.05)	0.07 (0.05)
**LEVEL 2**						
Self-concept (γ_010_)	−0.13^***^ (0.03)		−0.10^**^ (0.03)	−0.17^***^ (0.03)		−0.18^***^ (0.03)
Stereotype Endorsement [GSE] (γ_020_)		0.11^***^ (0.03)	0.07^*^ (0.03)		0.04 (0.03)	0.07^*^ (0.03)
Self-concept × GSE (γ_030_)						
**CROSS-LEVEL INTERACTIONS L1–L2**						
Trait × Self-concept (γ_110_)	−0.29^***^ (0.04)		−0.25^***^ (0.04)	−0.25^***^ (0.04)		−0.25^***^ (0.04)
Trait × GSE (γ_120_)		0.21^***^ (0.05)	0.12^*^ (0.05)		−0.09^+^(0.04)	−0.04 (0.04)
**VARIANCE COMPONENTS**						
Within-student (L1) variance (ơ^2^)	0.455	0.455	0.455	0.408	0.408	0.408
Intercept (L2) variance (τ_00_)	0.202	0.206	0.198	0.185	0.217	0.182
Slope (L2) variance (τ_11_)	0.047	0.090	0.034	0.045	0.102	0.043
Intercept-slope (L2) covariance (τ_01_)	−0.060	−0.043	−0.066	−0.064	−0.019	−0.062
Intercept (L3) variance	0.035	0.036	0.033	0.036	0.026	0.033
**Explanatory power**	0.649	0.328	0.746	0.583	0.056	0.602

*Unstandardized b coefficients are shown. Trait: 0 = state, 1 = trait; GSE = gender stereotype endorsement; female sample: N_Level 1_ = 3432; N_Level 2_ = 416; N_Level 3_ = 42; male sample: N_Level 1_ = 2775; N_Level 2_ = 339; N_Level 3_ = 42. Explanatory power refers to the proportion of slope variance explained by the L2 predictors (Aguinis et al., 2013). The slope variance of the model in which no cross-level interactions are included was τ_11_ = 0.134 for female sample and τ_11_ = 0.108 for male sample*.

* *p < 0.05*,

** *p < 0.01*,

*** *p < 0.001*,

+ *p < 0.10*.

Results indicated a significant trait-state discrepancy for girls (γ_100_ = 0.22, *p* < 0.01) that was not significant for boys (γ_100_ = 0.07, *ns*). As stated in Hypothesis 2, self-concept (Model 1) significantly and negatively predicted the trait-state discrepancy for girls (γ_110_ = −0.29, *p* < 0.001) as well as boys (γ_110_ = −0.25, *p* < 0.001). However, gender stereotype endorsement (GSE; Model 2) was found to differentially predict the trait-state discrepancy based on gender as stated in Hypothesis 3: This discrepancy was positively predicted by GSE for girls (γ_120_ = 0.21, *p* < 0.001), whereas for boys the effect of GSE was negative and marginally significant (γ_120_ = −0.09, *p* = 0.05).

When introducing both predictors to the model (Model 3), self-concept (γ_110_ = −0.25, *p* < 0.001) and GSE (γ_120_ = 0.12, *p* < 0.05) were both found to significantly predict the trait-state discrepancy for girls. For males, self-concept continued to be a significant predictor of the trait-state discrepancy (γ_110_ = −0.25, *p* < 0.001) but GSE was no longer a significant predictor (γ_120_ = −0.04, *ns*). For the sake of completeness, an additional interaction term between self-concept and gender stereotype endorsement was evaluated in a last step, but was not reported in the table as it did not significantly predict the trait-state discrepancy for girls or boys.

## Discussion

The aim of the present study was to shed light on gender differences in students' trait vs. state math anxiety. Furthermore, this study examined additional variables that were expected to contribute to the discrepancy between students' perceptions of trait math anxiety and their state math anxiety experiences in evaluating the effects of both students' self-concept and endorsement of a math-related gender stereotype as moderators of the trait-state discrepancy. Our study results replicate previous findings (Goetz et al., 2013) in showing girls to report higher levels of math anxiety relative to boys on trait assessments but not on state assessments of math anxiety with gender differences in trait anxiety being small in terms of effect sizes, but still significant.

Furthermore, self-concept negatively predicted the trait-state discrepancy as in previous studies (Goetz et al., 2013; Bieg et al., 2014), with our results additionally showing students' endorsement of a math-related gender stereotype to predict the trait-state discrepancy for female students. Girls who believed mathematics to be a male domain showed a larger discrepancy between their trait and state anxiety levels, with retrospective trait self-reports of anxiety being significantly higher than the state levels reported during actual math classes. This finding is in line with the assumption that gender stereotypes more strongly bias trait self-report measures than state measures (Robinson and Clore, 2002).

Our models also showed both self-concept and gender stereotype endorsement to predict trait-state discrepancy in female students when evaluated simultaneously. However, results showed the effect of stereotype endorsement to diminish when self-concept was introduced (see Model 3), suggesting that stereotype endorsement and self-concept are not entirely independent concepts. Nonetheless, it is important to note that stereotype endorsement was found to predict trait-state discrepancy over and above the effects of self-concept specifically for girls, highlighting the unique detrimental influence of these stereotyped mathematics beliefs on retrospective accounts of math anxiety primarily for female students.

Compared to female students, males were found to hold more stereotyped views of mathematics (see Hyde et al., 1990). Additionally, the effect of stereotype endorsement was inverted and marginally significant for male students, suggesting a tendency for male students who endorse the math-related gender stereotype to have lower trait-state discrepancies in their math anxiety. The negative implications of this mathematics-related gender stereotype for girls, and potential positive connotations for boys (cf. Steffens and Jelenec, 2011), confirm the expected pattern of results in showing a stronger overestimation of trait anxiety as compared to state anxiety for girls than for boys. In contrast, a strong mathematics self-concept, appears to serve a protective function for girls, particularly when performance-related gender stereotypes threaten their self-perceptions and emotional well-being in this domain. To summarize, our study findings show gender stereotype endorsement to increase the discrepancy between girls' assumed anxiety as compared to their actual anxiety levels (state anxiety) with endorsement of this stereotype found instead to diminish the trait-state discrepancy for boys—enhancing their self-perceptions regarding mathematics (Kurtz-Costes et al., 2008).

As mentioned above, the effect of stereotype endorsement was found to be weaker when self-concept was included as a second predictor—a finding consistent with previous research indicating a substantial degree of shared variance between math-related gender stereotypes and self-concepts in the domain of mathematics (Schmader et al., 2004; Kurtz-Costes et al., 2008). Regarding the direction of relationship between these constructs, gender stereotype endorsement may represent one reason why girls have lower self-concepts despite similar performance relative to boys. However, it is also possible that explicitly asking students about gender stereotypes may prompt them to draw conclusions based on their self-concepts in mathematics about how applicable these stereotypes are to themselves as a male or female student. Although the endorsement of the math-related gender stereotype is lower in female as compared to male students, our findings suggest that stereotype endorsement nonetheless has quite a negative effect on girls' trait anxiety ratings and perhaps also their attitudes regarding mathematics.

Concerning the study limitations, although the study findings suggest that state reports may be better able than trait questionnaires to capture emotions as they are experienced in daily life (Bolger et al., 2003), they nonetheless remain self-report measures and may produce different results as compared to other methods (e.g., behavioral observation, biometric indicators). Additionally, stereotype endorsement was assessed by use of a single straightforward item (“Mathematics is a male domain”). Future studies are recommended to replicate our findings with multi-item measures and to explore more specific elements of gender stereotypes, for example, with respect to the perceived value of the domain, possible careers afforded by math training, or the perceived proportion of female participation of the workforce in math domains (see Forgasz et al., 2004). Because of the mentioned limitations of self-reports and the tendency to deny beliefs in stereotypes when directly asked (Greenwald and Banaji, 1995), future studies could assess stereotype endorsement by the use of implicit measures (see Steffens et al., 2010). It can be assumed that the effect of implicitly measured gender-stereotype endorsement as predictor of the trait-state discrepancy may be even more pronounced. Our study sample was also limited to 9th and 10th graders in the highest track of the German school system (Gymnasium). Although these students typically represent those who subsequently enroll in university and obtain professional occupations with high responsibility, future studies are recommended to replicate our findings with a broader sample of students.

Finally, despite the study emphasis on measure-related differences in math anxiety for girls, it is critical to not overlook the importance of one's self-concept in mathematics as a predictor of the trait-state discrepancy in math anxiety for boys, especially those with lower self-concept levels. Thus, in addition to the study findings suggesting continued efforts to counteract the persistence of gender stereotypes to improve math anxiety for girls, these findings also warrant renewed interest in efforts to enhance self-concept beliefs for all students regarding mathematics given the significant and consistent benefits observed for both girls and boys in this domain.

In conclusion, these findings show an alarming effect of stereotype endorsement for girls who, due to inaccurate beliefs in gender differences in math ability, are at risk of believing they are more anxious than they report feeling in mathematics domains. These results therefore underscore the importance of initiatives on the part of schools, teachers, and parents to address and counteract gender stereotypes that may, in turn, help to correct the underrepresentation of females in mathematics-related careers.

### Conflict of interest statement

The authors declare that the research was conducted in the absence of any commercial or financial relationships that could be construed as a potential conflict of interest.
