# Supplementary material for: Gender stereotype endorsement differentially predicts girls' and boys' trait-state discrepancy in math anxiety
Source: Front Psychol. 2015 Sep 17;6:1404. doi: 10.3389/fpsyg.2015.01404 (PMC4585180; doi:10.3389/fpsyg.2015.01404)
Supplement: Supplementary file 1 [file Table1.PDF]

Appendix A

Table A1

Hierarchical linear regression models for complete sample with anxiety as dependent variable

	Complete sample							
	Model 1	Model 2	Model 3	Model 4	Model 5	Model 6	Model 7	Model 8
**Level 1**								
Intercept (γ_{000})	1.66***	1.67***	1.67***	1.66***	1.66***	1.66***	1.66***	1.67***
	(0.02)	(0.04)	(0.03)	(0.04)	(0.04)	(0.04)	(0.04)	(0.04)
Trait (γ_{100})	0.06	0.15***	0.15***	0.08	0.07	0.08	0.07	0.07
	(0.05)	(0.03)	(0.04)	(0.05)	(0.05)	(0.05)	(0.05)	(0.05)
**Level 2**								
Sex (γ_{010})	0.02			0.02	0.02	0.02	0.02	0.01
	(0.04)			(0.04)	(0.04)	(0.04)	(0.04)	(0.04)
Self-concept (γ_{010})		-0.15***		-0.15***		-0.17***		-0.18***
		(0.02)		(0.02)		(0.03)		(0.03)
Stereotype Endorsement [SE] (γ_{020})			0.08***		0.08***		0.04	0.07*
			(0.02)		(0.02)		(0.03)	(0.04)
Self-concept × Sex (γ_{030})						0.04		0.07+
						(0.03)		(0.04)
SE × Sex (γ_{030})							0.08+	0.01
							(0.05)	(0.04)
**Cross-level interactions L1-L2**								
Trait × Sex (γ_{110})	0.15*			0.14*	0.15*	0.14*	0.15*	0.15*
	(0.07)			(0.06)	(0.07)	(0.06)	(0.07)	(0.06)
Trait × Self-concept (γ_{110})		-0.27***		-0.27***		-0.25***		-0.25***
		(0.03)		(0.03)		(0.04)		(0.04)
Trait × SE (γ_{120})			0.08+		0.08*		-0.09*	-0.04
			(0.04)		(0.04)		(0.04)	(0.04)
Trait × Self-concept × Sex (γ_{110})						-0.04		-0.01
						(0.05)		(0.05)
Trait × SE × Sex (γ_{130})							0.30***	0.16**
							(0.06)	(0.06)

Note. Unstandardized *b* coefficients are shown. Trait: 0 = state, 1 = trait; $N_{\text{Level 1}} = 6207$; $N_{\text{Level 2}} = 755$; $N_{\text{Level 3}} = 42$.

* $p < .05$. ** $p < .01$. *** $p < .001$ + $p < .10$.
